# Combination of computed tomography angiography with coronary artery calcium score for improved diagnosis of coronary artery disease: a collaborative meta-analysis of stable chest pain patients referred for invasive coronary angiography

**DOI:** 10.1007/s00330-023-10223-z

**Published:** 2023-10-13

**Authors:** Mahmoud Mohamed, Maria Bosserdt, Viktoria Wieske, Benjamin Dubourg, Hatem Alkadhi, Mario J. Garcia, Sebastian Leschka, Elke Zimmermann, Abbas A. Shabestari, Bjarne L. Nørgaard, Matthijs F. L. Meijs, Kristian A. Øvrehus, Axel C. P. Diederichsen, Juhani Knuuti, Bjørn A. Halvorsen, Vladymir Mendoza-Rodriguez, Yung-Liang Wan, Nuno Bettencourt, Eugenio Martuscelli, Ronny R. Buechel, Hans Mickley, Kai Sun, Simone Muraglia, Philipp A. Kaufmann, Bernhard A. Herzog, Jean-Claude Tardif, Georg M. Schütz, Michael Laule, David E. Newby, Stephan Achenbach, Matthew Budoff, Robert Haase, Federico Biavati, Aldo Vásquez Mézquita, Peter Schlattmann, Marc Dewey, COME-CCT

**Affiliations:** 1 Department of Radiology, Charité - Universitätsmedizin Berlin, Berlin, Germany; 2 Radiology Department, Clinique Saint Augustin, 112-114 avenue d’Arès, 33000 Bordeaux, France; 3 Diagnostic and Interventional, Radiology University Hospital Zurich, University of Zurich, Zurich, Switzerland; 4 Department of Cardiology, Montefiore, University Hospital for the Albert Einstein College of Medicine, New York City, NY USA; 5 Department of Radiology, Kantonsspital St Gallen, St Gallen, Switzerland; 6 Department of Radiology, Modarres Hospital, Shahid Beheshti University of Medical Sciences, Tehran, Iran; 7 Department of Cardiology, Aarhus Universtity Hospital, Aarhus, Denmark; 8 Department of Cardiology, University Medical Centre Utrecht, Utrecht, Netherlands; 9 Department of Cardiology, Odense University Hospital, Odense, Denmark; 10 Turku University Hospital and University of Turku, Turku, Finland; 11 Department of Cardiology, Ostfold Hospital Trust, Grålum, Norway; 12 Department of Cardiology, National Institute of Cardiology and Cardiovascular Surgery, Havana, Cuba; 13 Department of Medical Imaging and Intervention, Linkou Chang Gung Memorial Hospital, College of Medicine, Chang Gung University, 333 Taoyuan City, Taiwan; 14 Department of Cardiology, Centro Hospitalar de Vila Nova de Gaia, Vila Nova de Gaia, Portugal; 15 Department of Internal Medicine, University of Rome Tor Vergata, Rome, Italy; 16 Department of Nuclear Medicine, University Hospital Zurich, Zurich, Switzerland; 17 Department of Radiology, Baotou Central Hospital, Inner Mongolia Province, Baotou, China; 18 Department of Cardiology, S Chiara Hospital, Trento, Italy; 19 HeartClinic Lucerne, Lucerne, Switzerland; 20 Montreal Heart Institute, Université de Montréal, Montréal, Canada; 21 Department of Cardiology, Charité - Universitätsmedizin Berlin, Berlin, Germany; 22 Centre for Cardiovascular Science, University of Edinburgh, Edinburgh, UK; 23 Department of Cardiology, Friedrich-Alexander University Erlangen-Nuremberg, Erlangen, Germany; 24 Department of Cardiology, Lundquist Institute at Harbor-UCLA, Torrance, CA USA; 25 Institute of Medical Statistics, Computer Sciences and Data Science, University Hospital of Friedrich Schiller University Jena, Jena, Germany; 26 Berlin Institute of Health, 10117 Berlin, Germany

**Keywords:** Computed tomography angiography, Coronary angiography, Coronary artery disease

## Abstract

**Objectives:**

Coronary computed tomography angiography (CCTA) has higher diagnostic accuracy than coronary artery calcium (CAC) score for detecting obstructive coronary artery disease (CAD) in patients with stable chest pain, while the added diagnostic value of combining CCTA with CAC is unknown. We investigated whether combining coronary CCTA with CAC score can improve the diagnosis of obstructive CAD compared with CCTA alone.

**Methods:**

A total of 2315 patients (858 women, 37%) aged 61.1 ± 10.2 from 29 original studies were included to build two CAD prediction models based on either CCTA alone or CCTA combined with the CAC score. CAD was defined as at least 50% coronary diameter stenosis on invasive coronary angiography. Models were built by using generalized linear mixed-effects models with a random intercept set for the original study. The two CAD prediction models were compared by the likelihood ratio test, while their diagnostic performance was compared using the area under the receiver-operating-characteristic curve (AUC). Net benefit (benefit of true positive versus harm of false positive) was assessed by decision curve analysis.

**Results:**

CAD prevalence was 43.5% (1007/2315). Combining CCTA with CAC improved CAD diagnosis compared with CCTA alone (AUC: 87% [95% CI: 86 to 89%] vs. 80% [95% CI: 78 to 82%]; *p* < 0.001), likelihood ratio test 236.3, df: 1, *p* < 0.001, showing a higher net benefit across almost all threshold probabilities.

**Conclusion:**

Adding the CAC score to CCTA findings in patients with stable chest pain improves the diagnostic performance in detecting CAD and the net benefit compared with CCTA alone.

**Clinical relevance statement:**

CAC scoring CT performed before coronary CTA and included in the diagnostic model can improve obstructive CAD diagnosis, especially when CCTA is non-diagnostic.

**Key Points:**

*• The combination of coronary artery calcium with coronary computed tomography angiography showed significantly higher AUC (87%, 95% confidence interval [CI]: 86 to 89%) for diagnosis of coronary artery disease compared to coronary computed tomography angiography alone (80%, 95% CI: 78 to 82%, p < 0.001).*

*• Diagnostic improvement was mostly seen in patients with non-diagnostic C.*

*• The improvement in diagnostic performance and the net benefit was consistent across age groups, chest pain types, and genders.*

**Supplementary Information:**

The online version contains supplementary material available at 10.1007/s00330-023-10223-z.

## Introduction

The coronary artery calcium (CAC) score is a prognostic marker for subsequent coronary events [1] and a diagnostic marker for the presence of obstructive coronary artery disease (CAD) [2]. The absence of CAC is associated with a low (< 5%) prevalence of obstructive CAD [3–5], and the presence of CAC increases the probability of obstructive CAD [6–10]. On the other hand, CAC scoring provides no information on non-calcified plaques or luminal stenosis, limiting its role in patient screening and risk stratification [11–13].

Coronary CT angiography (CCTA) is a safe non-invasive modality that serves as an accurate gatekeeper to invasive coronary angiography (ICA) in patients with stable chest pain and suspected CAD [3, 4]. The DISCHARGE trial has shown that, in patients referred for ICA because of stable chest pain who have an intermediate pre-test probability of CAD, there was no demonstrable difference between CCTA and ICA as the initial test, in preventing major adverse cardiovascular events, while the rate of major procedure-related complications was lower in the CCTA group [14]. The Collaborative Meta-Analysis of Cardiac CT (COME-CCT) consortium has shown that CCTA is highly accurate in diagnosing obstructive CAD when ICA is used as the reference standard [15]. Nevertheless, CCTA is limited by a non-diagnostic test rate that was as high as 10.4% in COME-CCT [15] and between 5 and 6.4% in patients with stable chest pain in several large clinical trials [14, 16, 17]. The non-diagnostic test result was defined at the patient level, according to the local standards of the original diagnostic accuracy studies included in the COME-CCT database, when it was not possible to exclude a significant stenosis of 50% or more in a vessel due to the underlying image quality of at least one vessel. The CAC score could possibly fill this diagnostic gap and provide useful supplementary information in patients with suspected CAD and stable chest pain.

Compared to the CAC score, CCTA was found to have higher diagnostic accuracy in diagnosing obstructive CAD in patients with stable chest pain in a study of Wieske et al. [18]. Nevertheless, the added value of combining CAC with CCTA is unknown. Thus, we sought to investigate the diagnostic performance of combined CCTA findings and CAC scores in comparison to CCTA findings alone using ICA as the reference standard.

## Methods

### Patients

Our study population was obtained from the COME-CCT original dataset [19], which is a collaborative meta-analysis of individual patient data evaluating the diagnostic performance of CCTA with invasive coronary angiography as the reference standard in stable chest pain patients. Both tests were performed on all patients and only those with available CAC scores were included in our analysis. We excluded patients with an unstable presentation, known CAD, coronary stents, or bypass grafts (Appendix Fig. 1). Data collection, data harmonization, reporting bias, and risk of bias assessment have been reported before [15]. Briefly, we contacted all corresponding authors for published and unpublished studies. Data harmonisation was performed by two independent readers who checked data quality and accuracy in comparison to aggregated published data. The risk of bias was assessed by two independent readers, who were not involved in data collection or harmonisation.

### ICA, CCTA, and CAC score

ICA was performed according to local standards at the study sites and was used as the diagnostic reference standard in this individual-patient data meta-analysis. Obstructive CAD was defined as a diameter reduction of at least 50% of the coronary artery lumen. Two- and three-dimensional CCTA post-processing was done by experienced investigators as previously reported [15]. A 50% stenosis of the coronary artery lumen was considered obstructive CAD by CCTA. The Agatston score was used to quantify the amount of CAC based on enhanced images obtained before the CCTA scan [20].

### Subgroup analysis

Subgroup analyses were carried out by gender, chest pain type, and age group. Three age groups were defined: less than 45 years of age, 45 to 65 years, and more than 65 years. These analyses were performed to evaluate the diagnostic performance of CAC plus CCTA compared to CCTA alone among those clinically important subgroups.

To replicate the clinical scenario, an additional model was developed by utilizing a cut-off value defined by the initial model (Appendix Table 3) to dichotomize the CAC values. This specific cut-off value determines the threshold at which performing CCTA offers negligible benefits, indicating that the false positive rate depending on this CAC cut-off value would be close to the non-diagnostic rate of CCTA. Additionally, when CCTA results are non-diagnostic, an additional CAC cut-off value of 400 is utilized.

### Statistical analysis

Normally distributed variables were reported as mean ± standard deviation (SD), while median and interquartile range (IQR) were used for ordinal or not normally distributed data. Categorical data were reported as proportions and their 95% confidence intervals (CI).

To evaluate the benefit of incorporating the CAC score and CCTA findings in the diagnosis of obstructive CAD, we constructed two prediction models for CAD: (1) the CCTA alone model and (2) the CAC plus CCTA model. The STARD 2015 guidelines [21] were followed for reporting the development and validation of the two models. Models were built based on an individual patient data (IPD) meta-analysis by generalized linear mixed effect models using a binomial distribution and a logistic link function and with a random intercept set for the individual studies to control for the variability between the 29 included studies. For the statistical comparison, the likelihood ratio test was used to compare the CCTA alone model and the CAC plus CCTA model. The diagnostic performance was compared based on the area under the receiver-operating-characteristic curve (AUC) [21–23] and the diagnostic odds ratio (DOR) [21], while the clinical consequences were assessed by a bias-corrected decision curve analysis [23]. Decision curve analysis is used to calculate net benefit across a range of threshold probabilities. The net benefit reflects the trade-off between the benefit of detecting new cases (true positive) and the harm of conducting the diagnostic test on patients who do not require it (false positive). The threshold probability pertains to the rate at which the clinical benefit and harm are reconciled. Bias correction for decision curve analysis was done by tenfold cross-validation with a 100 repeats approach [24]. The calibration of the models was assessed by calibration slope and visually by the calibration curve.

The intention-to-diagnose approach was followed to impute the CCTA results in case of non-diagnostic CCTA findings [25]. This is a worst-case scenario approach treating non-diagnostic CCTA cases as if they were false positive or false negative based on ICA results as the reference standard [25]. Model validation (Appendix Table 1) and calibration (Appendix Fig. 2) were done using 250 bootstraps [26]. Analyses were performed by R 4.2, using the packages lme4, pROC, reportROC, DescTools, PrediABEL, and dca-function (https://www.mskcc.org/departments/epidemiology-biostatistics/health-outcomes/decision-curveanalysis-01).

## Results

A total of 2315 patients from 29 studies were included in this analysis. The pre-test probabilities between the patients included in this analysis (0.52 ± 0.23) and the excluded patients (0.52 ± 0.22) showed no statistically significant difference (difference: 0.005 [95% CI: − 0.002 to 0.012], *p* = 0.153). Patients’ characteristics are summarized in Table 1. The study patients had a median CAC score of 71 (IQR: 1.0 to 378.3), and 1007 (43.5%) patients had obstructive CAD by ICA (Table 1). There was no statistically significant difference in CAD prediction based on the CAC score between patients with diagnostic CTA results and patients with non-diagnostic CTA results (AUC: 76% [95% CI: 74 to 78%] vs. 74% [95% CI: 67 to 80%], *p* = 0.529).

**Table 1:** Baseline characteristics

	Total (*n* = 2315)
Female	858 (36.5)
Age, yrs	61.1 ± 10.2
Pretest probability^a^, %	52.3 ± 22.86
Chest pain type	
Typical angina	987 (42.6)
Atypical angina	720 (31.1)
Non-anginal chest pain	453 (19.6)
Other chest discomfort	155 (6.7)
CAC score median (IQR)	71 (1.0–378.3)
BMI	26.8 ± 4.1
Diabetes	416 (18)
Hyperlipidemia	1095 (47.3)
Hypertension	12,256 (54.3)
Current smoker	665 (28.7)
CT examinations on scanners with ≤ 64 detector rows	2067 (89.3)
Non-diagnostic CTA (NDX)	253 (10.9)
Obstructive CAD^b^ by ICA	1007 (43.5)
Obstructive CAD^c^ by CCTA	1202 (51.9)

Values are *n* (%) or mean ± SD

*ICA* invasive coronary angiography; *CCTA* coronary computed tomography angiography; *CAD* coronary artery disease; *BMI* body mass index in kg/m^2^; *IQR* interquartile range

^a^ Pretest probability was calculated based on the Diamond-Forrester model and included gender, age, and type of symptoms

^b^ Obstructive CAD was defined as a diameter reduction of at least 50% on invasive coronary angiography (ICA) of the coronary artery and was used as the reference standard

^c^ Obstructive CAD was defined as a diameter reduction of at least 50% on coronary computed tomography angiography (CCTA) of the coronary artery

### Diagnostic performance evaluation

**Fig. 1:**
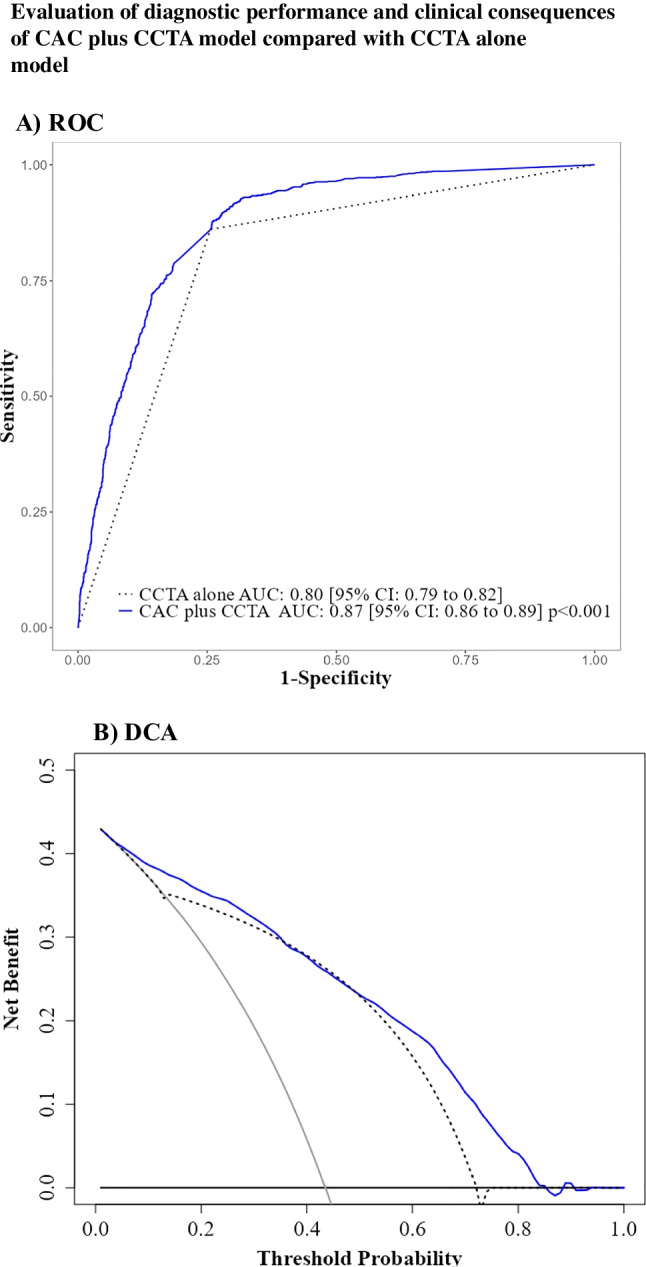
**A** Diagnostic performance assessed by the ROC curve shows that the CAC plus CCTA model (blue solid line) has a larger AUC than the CCTA alone model (dotted black line). **B** Clinical consequences by DCA show that the CAC plus CCTA (blue solid line) performed better than CCTA alone in terms of net clinical benefit (dotted black line). The black horizontal line represents the net benefit when all patients are considered to have no CAD, whereas the grey line represents the net benefit when all patients are considered to have obstructive CAD. The intersection of the two lines indicates the prevalence of obstructive CAD (43.3%) in our sample

Adding the CAC score to CCTA improved the AUC of CAD prediction to 87% [95% CI: 86 to 89%]) compared to CCTA alone (AUC: 80% [95% CI: 78 to 82%]) (*p* < 0.001) (Fig. 1A), likelihood ratio test 236.3, df: 1, *p* < 0.001*.* The other diagnostic performance parameters for the two models are compiled in the Supplement (Appendix Table 2). The CAC score plus the CCTA model improved the diagnostic accuracy by 2.4% (*p* < 0.001) by correctly classifying 55 net cases. Most of these cases, 52 (96%), were patients with non-diagnostic CCTA results. The remaining three cases (4%), which showed typical symptoms and had CAC scores exceeding 2000, were patients with diagnostic CCTA results. Appendix Tables 3–4 present the estimates for both models and the result of the likelihood ratio test for their comparison.

The DOR of the CAC score plus CCTA model was 64.0 [95% CI: 35.9 to 114.2], compared with, the CCTA alone model 41.3 [95% CI: 20.4 to 83.5] (Appendix Figs. 3−4).

### Evaluation of clinical consequences

Bias-corrected decision curve analysis showed that the CAC score plus CCTA model performed better than CCTA alone in terms of net benefit over almost all threshold probabilities (Fig. 1B).

### Subgroup analysis

**Fig. 2:**
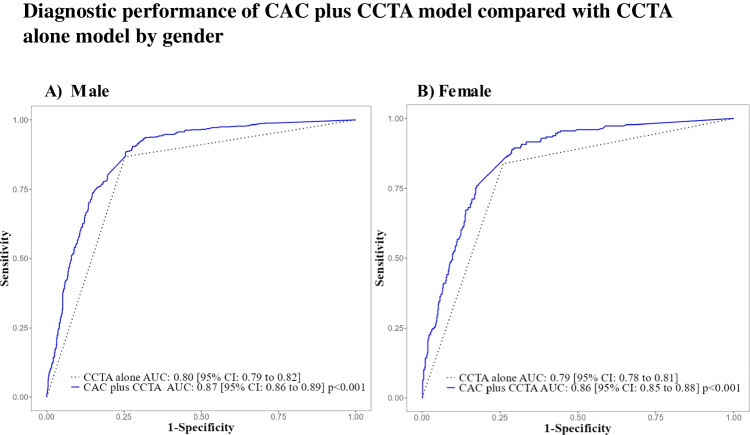
ROC curve subgroup analysis by gender shows a consistently better diagnostic performance for the CAC plus CCTA model (blue solid line) over the CCTA alone model (dotted black line) among (**A**) male and (**B**) female patients

**Fig. 3:**
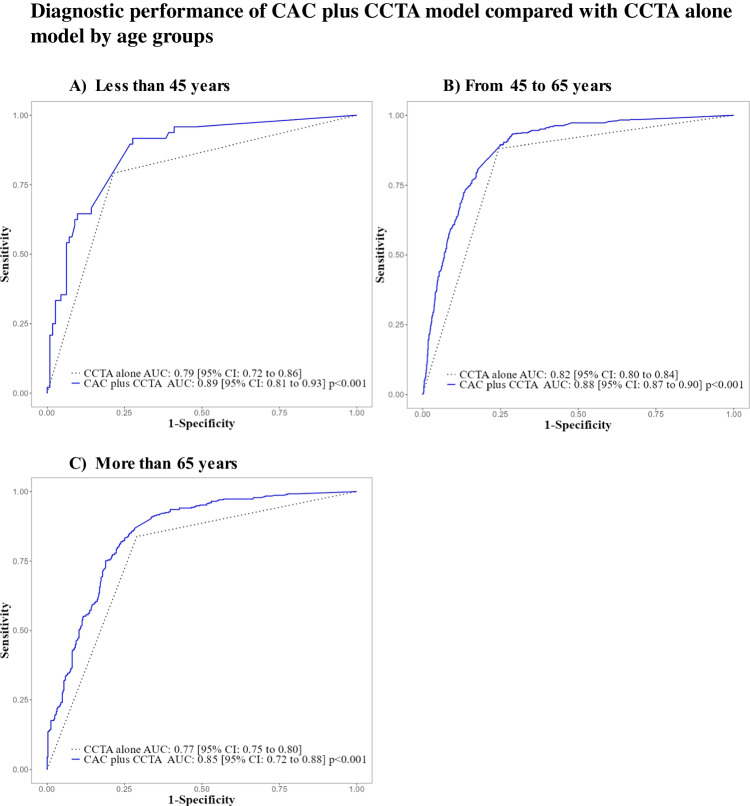
ROC curve subgroup analysis by age groups shows a consistently better diagnostic performance for the CAC plus CCTA model (blue solid line) over the CCTA alone model (dotted black line) across all three age groups investigated

**Fig. 4:**
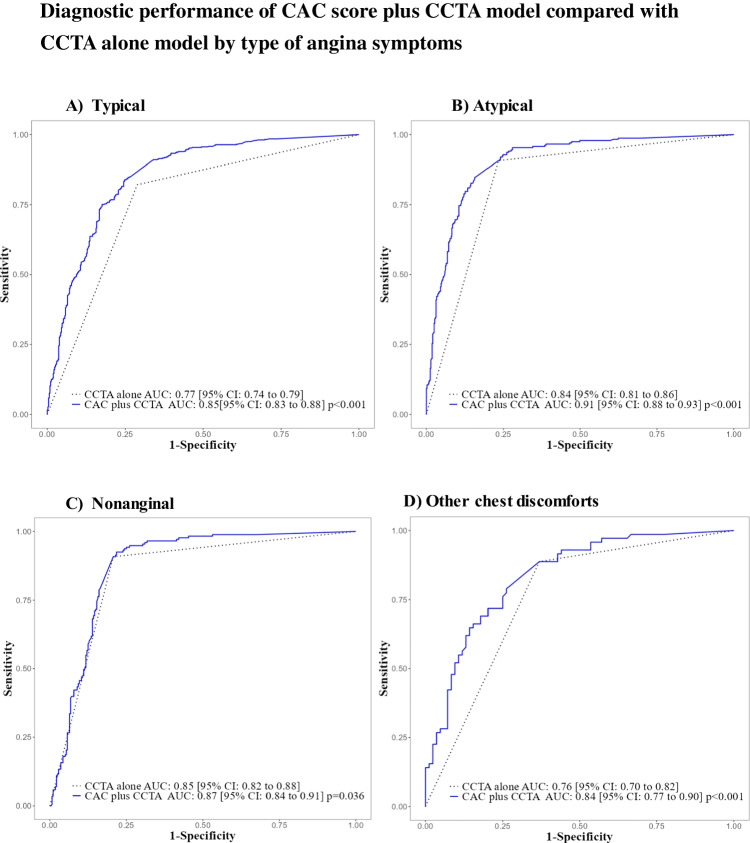
ROC curve subgroup analysis by type of chest symptoms shows a consistently better diagnostic performance for the CAC plus CCTA model (blue solid line) over the CCTA alone model (dotted black line) across all chest pain types

Combining the CAC score with CCTA findings improved the AUC of obstructive CAD diagnosis in both (Fig. 2), all three age groups (Fig. 3), and across types of angina symptoms (Fig. 4), when compared to CCTA alone.

**Fig. 5:**
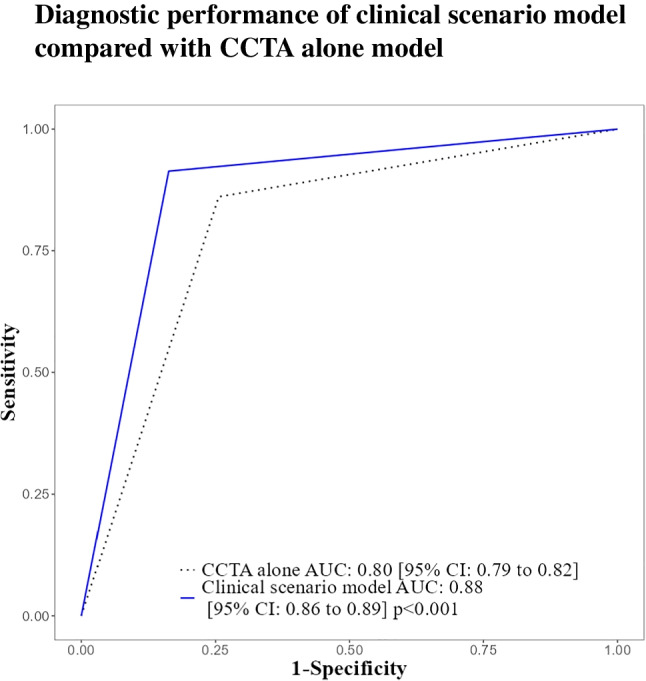
ROC curve of the clinical scenario model (blue solid line) based on two CAC cut-off values. The first cut-off value of 2000 is used to directly refer patients to ICA, while the second cut-off value of 400 is used in the case of non-diagnostic CCTA. The clinical model (blue solid line) has a larger AUC than the CCTA alone model (dotted black line)

The initial model suggested that it may be legitimate to directly refer patients with a CAC score of 1715 or higher to ICA. However, for the sake of simplicity, a CAC cut-off value of 2000 was used in our clinical scenario model. The clinical scenario model showed an improved CAD prediction with an AUC of 88% [95% CI: 86 to 90%], compared to CCTA alone with an AUC of 80% [95% CI: 78 to 82%] (*p* < 0.001). Additionally, the clinical scenario model significantly enhanced the diagnostic accuracy to 87% (95% CI: 86 to 88%), compared to CCTA alone with an accuracy of 80% (95% CI: 78 to 81%) (*p* < 0.001) (Fig. 5).

## Discussion

Our analysis yielded four major findings: First, Combining the CAC score with CCTA findings improved obstructive CAD prediction compared to CCTA alone in patients with suspected CAD and stable chest pain. Second, the combination of the CAC score with CCTA showed a higher clinical benefit over nearly the entire range of threshold. Third, a better calibration curve and higher DOR were found for the CAC score plus CCTA model compared with the CCTA alone model, which may suggest better external validity in different settings, especially in patients with lower CAD prevalence. Finally, we also showed that the CAC score plus CCTA findings showed better performance than CCTA alone among the different clinically important subgroups (gender, age, type of chest pain). These findings can be explained by the ability of CAC to fill the diagnostic gap of CCTA in patients with non-diagnostic test results for the diagnosis of CAD.

Our CAD prediction model is based on two pillars: CCTA findings and the CAC score.

To our knowledge, four clinical trials compared CCTA with ICA, the single-centre CAT-CAD trial [27], the multicentre CONSERVE trial [28], the single-centre randomised CAD-Man trial [29], and the multicentre DISCHARGE trial [14]. A recent meta-analysis of the four trials showed that CCTA and ICA have a similar predictive ability of major adverse cardiovascular events at a median follow-up of 2.8 years [30]. However, none of the previously mentioned trials examined the combination of CAC score and CCTA findings.

The simplicity of the CAC score, the avoidance of contrast medium as well as low radiation requirements make it an applicable diagnostic marker for the detection of CAD. While it has already been shown that adding the CAC score to a patient’s cardiovascular risk factors enhances the pre-test probability prediction of CAD, the 2019 European guidelines did not recommend the routine use of the CAC score in CAD diagnosis despite its net reclassification improvement of 66% [3]. In contrast, the 2021 American College of Cardiology guidelines state that CAC scores, if available, can be used as a first-line test for better estimation of the pre-test probability [4]. For patients with an intermediate to high risk of CAD and non-diagnostic CCTA findings, the American College of Cardiology guidelines recommend that CAC score testing should be added to stress testing [4], while it is not clear if adding a CAC score to CCTA findings is of any benefit.

We showed that combining the CAC score with CCTA findings improved the diagnostic performance in detecting obstructive CAD in comparison to CCTA alone, which was also reflected in a better net benefit over almost all threshold values for the combined tests, which will improve clinical decision-making in the different clinical scenarios. Furthermore, the improvement in the diagnostic performance was consistent across all clinically important subgroups, especially in patients with typical chest pain symptoms and patients older than 65 years. Finally, obtaining information on CAC scores in the routine diagnostic workup of suspected CAD could contribute to a better assessment of the patient’s prognosis and better adjustment of the therapeutic regimen.

Furthermore, another algorithm was studied in the multicentre CRESCENT Trial, where CAC scoring was done first with the sequential addition of CCTA if CAC was positive, demonstrating that fewer patients randomized to cardiac CT reported anginal complaints (*p* = 0.012). After 1.2 years, event-free survival was 96.7% for patients randomized to CT and 89.8% for patients randomized to functional testing (*p* = 0.011). CT afforded faster diagnosis (*p* < 0.0001), and additional downstream testing was required less frequently (25 vs. 53%, *p* < 0.0001), resulting in lower diagnostic costs (€369 vs. €440; *p* < 0.0001) [31].

### Limitations

This study has relevant limitations. First, the COME-CCT study population was collected from different prospective diagnostic studies conducted to compare CCTA with ICA for the diagnosis of CAD but not for specifically investigating the additional value of CAC scoring. Second, CAC scores were not available for all patients included in the COME-CCT consortium. Third, patients included were referred for ICA, which resulted in a rather high prevalence of CAD (43.5%); therefore, external validation of our model in patient populations with lower CAD prevalence is warranted. Fourth, the analysis was done at the patient level only as no segment-wise information was available. Leschka et al. have shown that CAC scoring did not improve diagnostic accuracy when combined with segmental CCTA in all patients, whereas, in the subset of patients with non-diagnostic segments, specificity increased from 87 to 100% while sensitivity was not affected [32]. Fifth, the gender continuous variable was not used in the data collection of the originally included studies, which only used binary gender definitions.

Finally, there is no information on medications, especially statins, taken by our study patients, which would have been relevant because several studies have shown that statins increase the calcified plaque volume (NCPV) [33, 34].

### Strengths

This study also has strengths such as its large size including 29 original studies from 16 countries with an overall number of more than 2000 patients with CAC, CCTA, and ICA. Second, calibration assessment helped make our results generalizable to other settings with a lower prevalence of CAD. Third, ICA referral was essential to avoid verification bias, which has an unequivocal impact on the validity of the calibration measures. The use of CCTA as a reference standard would have reduced the validity of model calibration, due to its low positive predictive value.

## Conclusion

Using ICA as the reference standard, this study shows that combining CAC scores with CCTA findings improved the diagnostic performance and the net benefit in identifying obstructive CAD diagnosis compared with CCTA findings. This potential of CAC scores to fill the diagnostic gap, especially in patients with non-diagnostic CCTA, may lead to improved clinical decision-making in patients with stable chest pain. 

### Supplementary Information

Below is the link to the electronic supplementary material.

Supplementary file1 (PDF 744 KB)

## Abbreviations

AUC: Area under the receiver-operating-characteristic curve
CAC: Coronary artery calcium
CAD: Coronary artery disease
CCTA: Coronary computed tomography angiography
CI: Confidence interval
COME-CCT: Collaborative Meta-Analysis of Cardiac CT
DCA: Decision curve analysis
ICA: Invasive coronary angiography
IPD: Individual participant data
IQR: Interquartile range
NPV: Negative predictive value
PPV: Positive predictive value
SD: Standard deviation
DOR: Diagnostic odds ratio

## Declarations

### Guarantor

The scientific guarantor of this publication is Marc Dewey.

### Statistics and biometry

Two of the authors have significant statistical expertise.

### Study subjects or cohorts overlap

This is a sub-analysis of the CoMe-CCT main cohort (5332 patients), published previously by Haase et al (2019), https://doi.org/10.1136/bmj.l1945. Another sub-analysis of 2452 patients after the exclusion of 2880 patients (2878 patients without an available CAC score and 2 patients due to data inconsistency), Wieske et al (2022), https://doi.org/10.1007/s00330-022-08619-4.

### Methodology

• prospective

• diagnostic or prognostic study

• multicentre study
