# Supplementary material for: Combination of computed tomography angiography with coronary artery calcium score for improved diagnosis of coronary artery disease: a collaborative meta-analysis of stable chest pain patients referred for invasive coronary angiography
Source: Eur Radiol. 2023 Oct 13;34(4):2426–36. doi: 10.1007/s00330-023-10223-z (PMC10957619; doi:10.1007/s00330-023-10223-z)
Supplement: Supplementary file 1 — Supplementary file1 (PDF 744 KB) [file 330_2023_10223_MOESM1_ESM.pdf]

Combination of computed tomography angiography with coronary artery calcium score for improved diagnosis of coronary artery disease: a collaborative meta-analysis of stable chest pain patients referred for invasive coronary angiography

Electronic Supplementary Material

Eur Radiol (2023) Mohamed M, Bosserdt M, Wieske V et al.

APPENDIX RESULTS

Appendix Figure 1: Flowchart

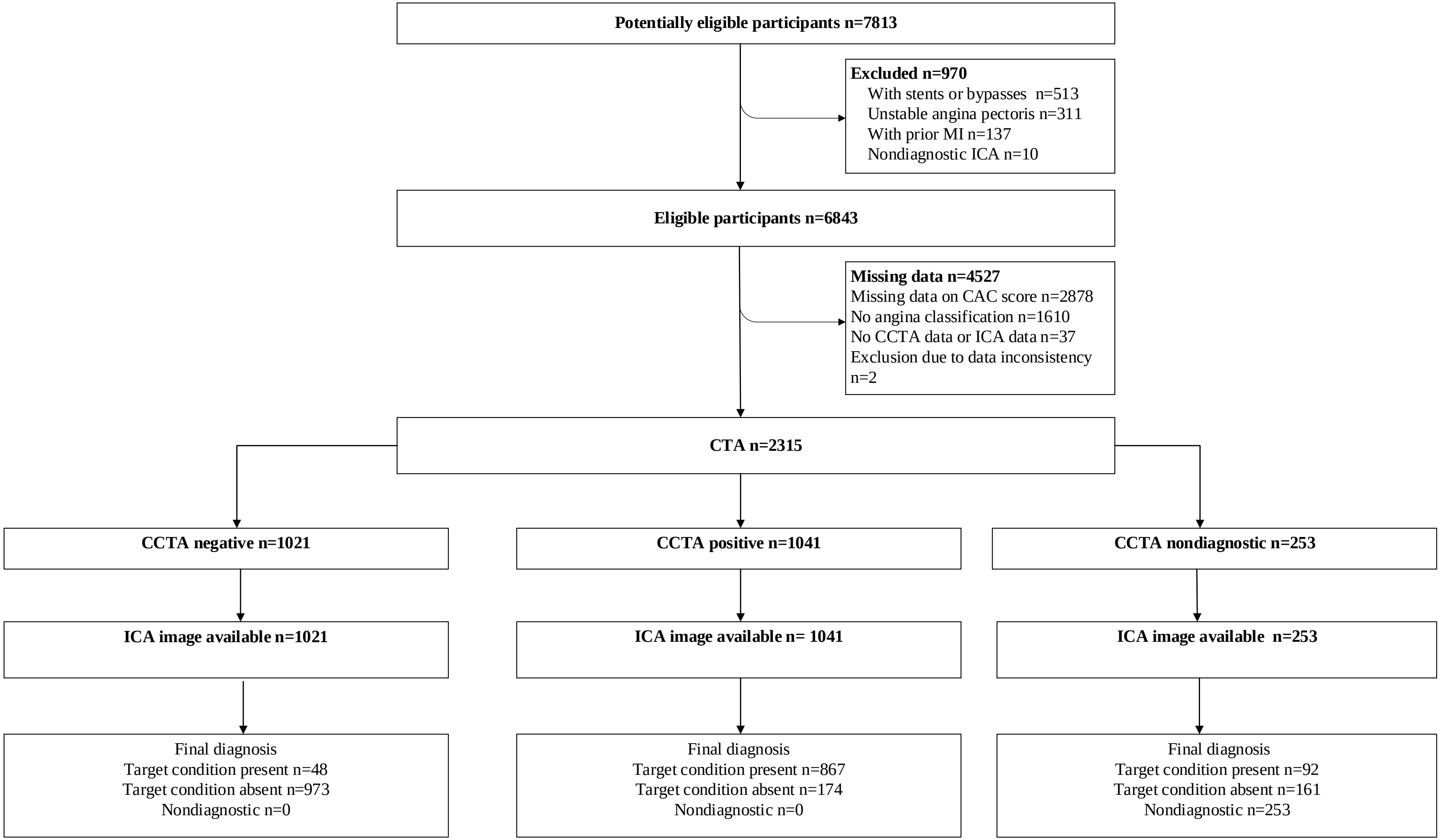

Overall, 2315 patients with complete data on age, gender, chest pain type, and CAC score from 29 studies were included from the CoMe-CCT dataset (7813 patients from 76 studies)

Appendix Figure 2: Calibration plot of the CAC-plus-CTA model and CTA-alone model

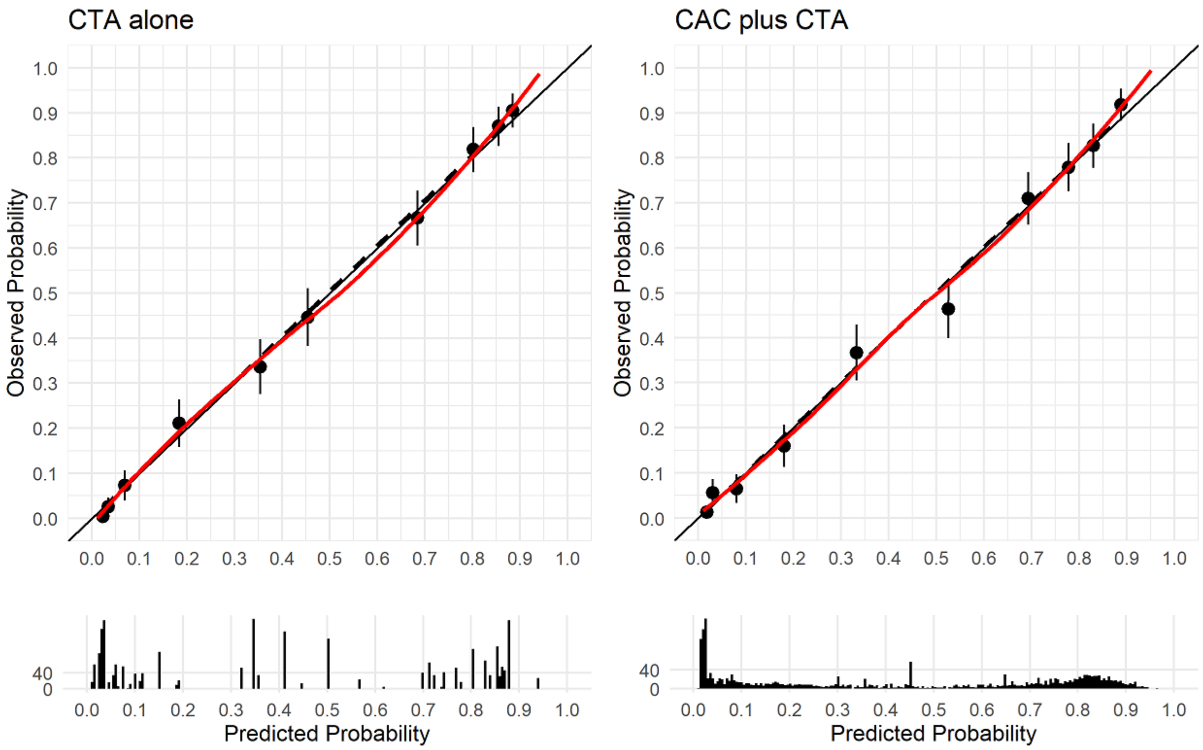

Appendix Figure 3: Forest plot of the CTA-alone model.

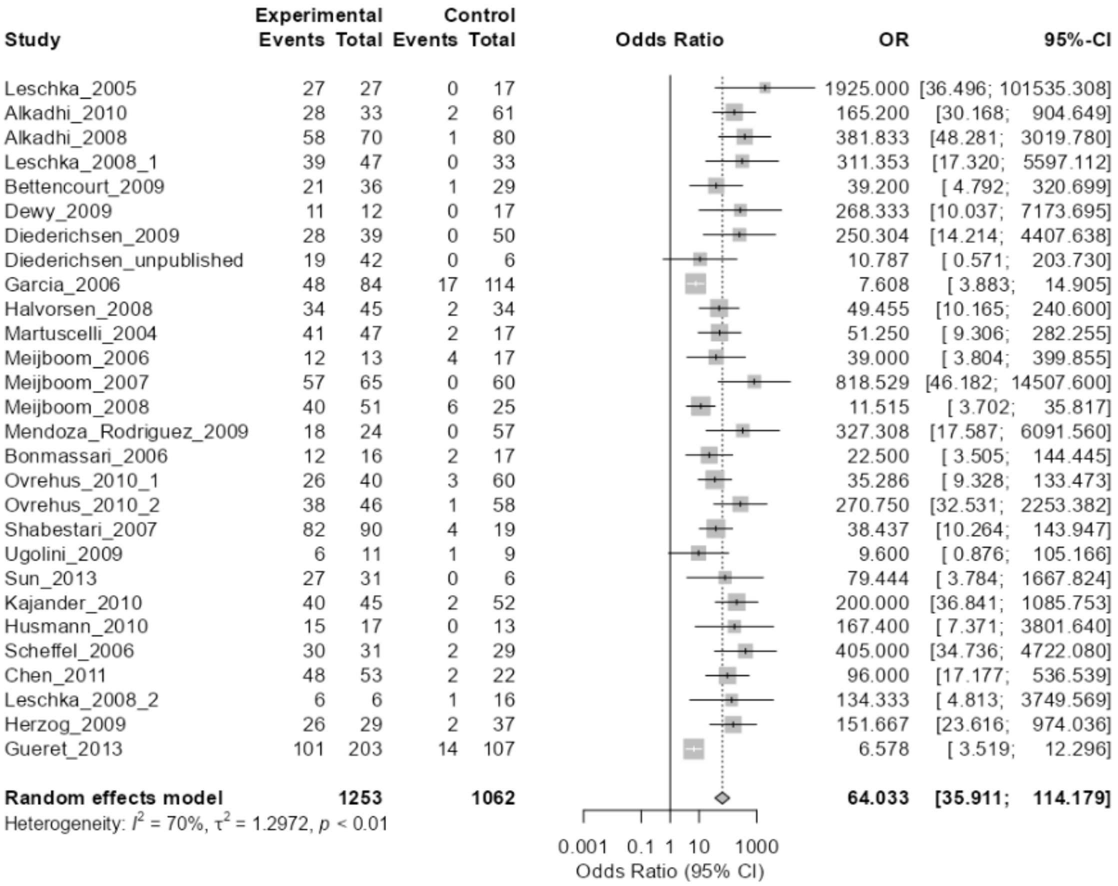

Appendix Figure 4: Forest plot of the CAC-plus-CTA model.

Study	Experimental Events	Experimental Total	Control Events	Control Total	OR	95%-CI
Leschka_2005	27	27	0	17	1925.000	[36.496; 101535.308]
Alkadhi_2010	28	33	2	61	165.200	[30.168; 904.649]
Alkadhi_2008	58	70	1	80	381.833	[48.281; 3019.780]
Leschka_2008_1	39	47	0	33	311.353	[17.320; 5597.112]
Bettencourt_2009	21	36	1	29	39.200	[4.792; 320.699]
Dewy_2009	11	12	0	17	268.333	[10.037; 7173.695]
Diederichsen_2009	28	39	0	50	250.304	[14.214; 4407.638]
Diederichsen_unpublished	19	42	0	6	10.787	[0.571; 203.730]
Garcia_2006	48	84	17	114	7.608	[3.883; 14.905]
Halvorsen_2008	34	45	2	34	49.455	[10.165; 240.600]
Martuscelli_2004	41	47	2	17	51.250	[9.306; 282.255]
Meijboom_2006	12	13	4	17	39.000	[3.804; 399.855]
Meijboom_2007	57	65	0	60	818.529	[46.182; 14507.600]
Meijboom_2008	40	51	6	25	11.515	[3.702; 35.817]
Mendoza_Rodriguez_2009	18	24	0	57	327.308	[17.587; 6091.560]
Bonmassari_2006	12	16	2	17	22.500	[3.505; 144.445]
Ovrehus_2010_1	26	40	3	60	35.286	[9.328; 133.473]
Ovrehus_2010_2	38	46	1	58	270.750	[32.531; 2253.382]
Shabestari_2007	82	90	4	19	38.437	[10.264; 143.947]
Ugolini_2009	6	11	1	9	9.600	[0.876; 105.166]
Sun_2013	27	31	0	6	79.444	[3.784; 1667.824]
Kajander_2010	40	45	2	52	200.000	[36.841; 1085.753]
Husmann_2010	15	17	0	13	167.400	[7.371; 3801.640]
Scheffel_2006	30	31	2	29	405.000	[34.736; 4722.080]
Chen_2011	48	53	2	22	96.000	[17.177; 536.539]
Leschka_2008_2	6	6	1	16	134.333	[4.813; 3749.569]
Herzog_2009	26	29	2	37	151.667	[23.616; 974.036]
Gueret_2013	101	203	14	107	6.578	[3.519; 12.296]
**Random effects model**		**1253**		**1062**	**64.033**	**[35.911; 114.179]**

Heterogeneity: $I^2 = 70\%$, $\tau^2 = 1.2972$, $p < 0.01$

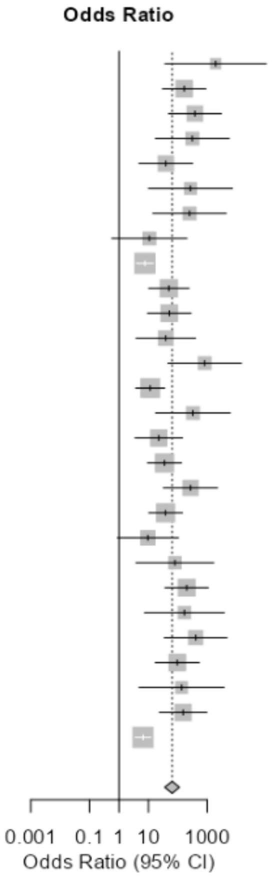

Appendix Table 1. Internal validation for CAC-plus-CTA model and CTA-alone model					
**Performance Measure**	**Original Model**	**Bootstrap Sample**	**Original Sample**	**Optimism**	**Bootstrap corrected**
**CTA-alone model**					
AUC[a]	80.2%	80.4%	80.4%	0.0000	80.2%
Brier Score[b]	0.149	0.149	0.149	0.0002	0.149
Scaled Brier Score[c]	0.4	0.4	0.4	0.00	0.4
Discrimination Slope[d]	0.400	0.397	0.396	0.000	0.399
Calibration Slope[e]	0.994	0.982	0.994	-0.0117	1.006
**CAC-plus-CTA model**					
AUC[a]	87.2%	87.2%	87.1%	-0.00002	87.2%
Brier Score[b]	0.132	0.132	0.132	0.000	0.132
Scaled Brier Score[c]	0.47	0.47	0.47	0.0001	0.47
Discrimination Slope[d]	0.455	0.454	0.455	-0.0013	0.456
Calibration Slope[e]	0.984	0.971	0.985	-0.0136	0.998

[a] AUC: area under the receiver-operating-characteristic curve as a concordance statistics
[b] Brier score: measuring the accuracy of probabilistic predictions by quantiyfing the prediction error. A perfect model has a zero Brier score
[c] Scaled Brier score: is scaled by its maximum score and accounts for the outcome (CAD) prevalence. A perfect model has a score of 1
[d] Discrimination slope: is the difference in mean of predicted probabilities between disease (CAD) and no disease (no CAD)
[e] Calibration slope: measures the agreement between the actual outcome and predicted probabilities.

Appendix Table 2. Diagnostic Performance of CAC-plus-CTA and CTA-alone model			
	**CAC plus CTA** *n/total n (% [95% CI*])*	**CTA alone** *n/total n (% [95% CI*])*	**p-value***
Diagnostic accuracy	1895 / 2315 (81.9% [81.7-82.0])	1840 / 2315 (79.5% [79.4-79.7])	<0.0001
Sensitivity	846/1007 (83.9% [81.6-86.2])	867 / 1007 (86.1% [84.0-88.2])	<0.021
Specificity	1050 / 1308 (80.3% [78.1-82.4])	973/1308 (74.4% [72.0-86.8])	<0.0001
Positive predictive value	846 / 1103 (76.6% [74.1-79.1])	867/1202 (72.1% [69.6-74.7])	<0.0001
Negative predictive value	1050/ 1212 (86.6% [84.7-88.5])	973/1113 (87.4% [85.5-89.4])	0.257

**Appendix Table 3. Estimates of the mixed-effect logistic regression model of CTA-alone and CAC-plus-CTA models (n=2315)**			
	**Estimate (S.E.)**	**p value**	**Odds Ratio (95% CI)**
**CTA-alone model**			
CTA	2.91 (0.116)	< 0.001	18.3 (14.63 to 23.03)
**Model constant**	-1.897 (0.124)	—	—
**Random intercept (τ2)** [b]	0.167(0.411)		
BIC	2256.9		
logLik	-1116.8		
**CAC-plus-CTA model**			
Log CAC score[c]	0.362 (0.026)	< 0.001	1.44 (1.37 to 1.51)
CTA	2.67 (0.123)	< 0.001	14.51 (11.42 to 18.54)
**Model constant**	-3.257 (0.183)	—	—
**Random intercept (τ2)** [b]	0.272 (0.522)		
BIC	2028.4		
logLik	-998.7		

BIC: Bayesian information criterion

[a]Typical angina is defined as: retrosternal chest discomfort, precipitation by exertion, and prompt relief (within 30 s-10 min) by rest or nitroglycerin. Patients in whom two, one, or none of these three criteria were found were classified as having atypical angina, nonanginal chest discomfort, and other chest discomfort, respectively.

[b]Variance component estimate (τ2) for random intercept

[c]Log CAC score was used to overcome its nonnormality.

CAC cut-off value = $e^{(\beta_{CTA}/\ \beta_{log\ CAC})}$ = 1715

**Appendix Table 4. Likelihood ratio test comparing the CTA-alone and CAC-plus-CTA models (n=2315)**					
	**BIC**	**logLik**	**Deviance**	**X^2**	**p value**
**CTA alone**	2256.9	-1116.8	2233.7		
**CAC plus CTA**	2028.4	-998.7	1997.4	236.25	<0.001
